# Outbreak of diarrheal diseases causes mortality in different geographical locations of Bangladesh during the 2021 COVID-19 era

**DOI:** 10.3389/fpubh.2023.1103518

**Published:** 2023-01-27

**Authors:** Ashraful Islam Khan, Md. Taufiqul Islam, Mohammad Ashraful Amin, Zahid Hasan Khan, Firdausi Qadri

**Affiliations:** ^1^International Centre for Diarrhoeal Disease Research, icddr, b, Dhaka, Bangladesh; ^2^School of Medical Science, Griffith University, Gold Coast, QLD, Australia

**Keywords:** Bangladesh, cholera, diarrhea, outbreak, COVID-19

## Abstract

**Objectives:**

Diarrhea is a major public health problem in low- and middle-income countries, including Bangladesh. Of the different spectrums of diarrheal diseases, cholera occurs every year, causing outbreaks and epidemics following a biannual seasonal pattern. Due to the COVID-19 pandemic, hospitalization for diarrheal diseases decreased in 2020 compared to the previous years. However, in 2021, massive outbreaks occurred in different geographical locations of the country. We described that an outbreak of diarrheal diseases causes mortality in different geographical locations in Bangladesh.

**Method:**

In this study, we present a report of diarrhea outbreaks that were reported in 2018–2021 in different parts of Bangladesh, and data have been captured from different sources such as print and electronic media as well as from a nationwide surveillance system.

**Results:**

Among these locations, districts of Barisal Division, Kishorganj, Noakhali, Gopalganj, Bandarban, and Chattogram were the major hotspots of the outbreaks where high morbidity due to acute watery diarrhea and even mortality, which is usually low in Bangladesh, were recorded.

**Conclusion:**

Early detection and prevention and strengthening of the surveillance system are needed to combat the diarrheal upsurge, take immediate control, and adopt preventive strategies.

## Introduction

Diarrhea is a disease of the gastrointestinal tract characterized by frequent, loose, and watery bowel movements. The etiology of the disease may be bacterial (*Vibrio cholerae*, ETEC, *Shigella*, and *Salmonella* spp.), viral (most commonly rotavirus), protozoa, and parasitic organisms, which can be spread by contaminated water (1). Diarrheal diseases have been considered a major public health problem and are estimated as the eighth leading cause of mortality globally (2). Most burden estimations have been focused on children due to their high prevalence in under five children (1.7 billion episodes annually among under five children) even though a substantial burden is seen in adults (2, 3). Overall deaths due to diarrhea have been reduced after the invention of oral rehydration solution (ORS), but morbidity has remained relatively constant (4). In Bangladesh, diarrheal diseases are the most common cause for seeking hospital-level care (5, 6). Along with over 210 countries globally, Bangladesh is also facing a large outbreak of COVID-19 at present. The World Health Organization (WHO) has declared it a pandemic emergency, and the first-ever COVID-19 case was detected in Bangladesh on 8 March 2020 (7). Due to the COVID-19 pandemic, hospitalization for diarrheal diseases decreased in 2020 compared to that in previous years. However, in 2021, massive outbreaks occurred in different geographical locations of the country. Epidemics and outbreaks are common in the region where there is a shortage of clean water for drinking, cooking, and cleaning and also among people with a lack of knowledge on basic hygiene and sanitation. Most importantly, water contaminated with feces from municipal sewage, septic tanks, and latrines is the cause of disease outbreaks. However, water scarcity, increased salinity of water, and climate change are the predisposing factors for increased diarrheal disease outbreaks. Monitoring outbreaks can help us learn more about the causes of outbreaks, sources, and the groups of people who become ill. This knowledge can be used to control the outbreak and prevent the further spread or recurrence of the infection in future. Diarrheal diseases including cholera outbreaks occurred in Bangladesh many times, including during floods (1988, 1998, and 2004) and due to behavioral factors such as using contaminated sources of water and poor hygiene practices (8, 9). Cholera is an extremely virulent disease that can cause severe acute watery diarrhea, and the WHO has launched an aim to end cholera by 2030, with the major target being to eliminate cholera from 20 countries out of 47 cholera-endemic countries by 2030 (10). To align with this target, burden estimation, following the disease trends, integrated disease surveillance, and rapid outbreak investigation are crucial. The reason behind this may have been the fear of seeking treatment in hospitals, maintaining good hand-washing practices, and avoiding street food due to the COVID-19 pandemic. However, from April 2021, the country experienced sporadic outbreaks of diarrheal diseases in different districts, causing a huge number of cases and deaths. In this report, a brief update on this upsurge/outbreak of diarrheal diseases including cholera is described based on reports from different media.

## Method/data source

Information on diarrheal outbreaks was collected from different print and electronic media as well as personal communication with different hospitals and institutes around the country. We adopted various electronic data sources for this report, which include newspaper and television news broadcasts. Very limited data on the microbiological cause of outbreaks were available. However, nationwide sentinel cholera surveillance has been continuing in 16 sites in Bangladesh in collaboration between icddr,b and the Institute of Epidemiology Disease Control and Research (IEDCR) (11). In this surveillance, participants with acute watery diarrhea have been enrolled, and also a rapid diagnostic test (RDT) and microbiological culture have been carried out for collected samples. We have also used the data from this surveillance network for this outbreak report.

## Result and discussion

Barisal Division, located in south-central Bangladesh, has been affected by diarrheal diseases since the beginning of this year. According to one report by a local newspaper, over 50,000 patients received treatment for diarrheal diseases in health facilities between 1st January and 10th May 2021. According to government records, 19 patients have died whereas non-government sources have recorded 36 deaths (12). Between 1st April and 23rd April, a total of 38,046 cases and 10 deaths were reported, with the highest number of cases in Bhola and the highest number of deaths in the Barisal district in Bangladesh's Barisal division (Table 1). In addition, an average of more than 1,000 people was admitted to hospitals each day in mid-April 2021. Considering this critical situation, a national ‘outbreak response' from the Institute of Epidemiology Disease Control and Research (IEDCR) was carried out. The groups worked in these areas to investigate the burden and etiology of these upsurges. After laboratory testing, *Vibrio cholerae* O1, *E. coli*, and other bacteria were found in the stool sample of the affected patients. It was assumed that contaminated water and other environmental factors, such as scarcity and increased salinity of water, were the sources of infection. Usually, the people in these areas used tube well for drinking water but for other household purposes such as cooking and washing, they used water from natural sources (rivers and ponds). Furthermore, people are used to eating “Panta” (cooked rice soaked in water overnight or longer), and for this preparation, they use water from natural sources. Government officials have warned the public that the magnitude of diarrhea and deaths caused by AWD diarrhea in Barisal has surpassed previous two-decade records (13, 14). The Ministry of Health and Family Welfare (MOH&FW) took immediate action to combat this epidemic by ensuring medicines and saline and by taking some action of awareness. The nationwide sentinel surveillance covers three outbreak areas (Barisal, Patuakhali, and Pirojpur). During the outbreak in April 2021, a total of 147 samples were collected from these areas. Among the tested sample, 28% (*n* = 41) was RDT positive, and also 16% (*n* = 23) of the culture-confirmed organism has been isolated from stool samples for *Vibrio cholerae* O1.

**Table 1:** Number of cases and deaths due to diarrheal disease outbreaks in different districts/upazila in Bangladesh, 2021.

**District**	**Case**	**Death**
Bhola	9,355	00
Barisal	4,989	05
Patuakhali	6,290	03
Pirojpur	4,204	00
Barguna	5,634	02
Jhalokati	3,797	00
Bandarban/ Alikadam	136	06
Kishoreganj/Mithamoin	100	04
Gopalganj	1,563	00
Noakhali	10,000	15
Bhasan Char (Hatiya)	1,500	04
**Total**	**47,568**	**39**

**Figure 1:**
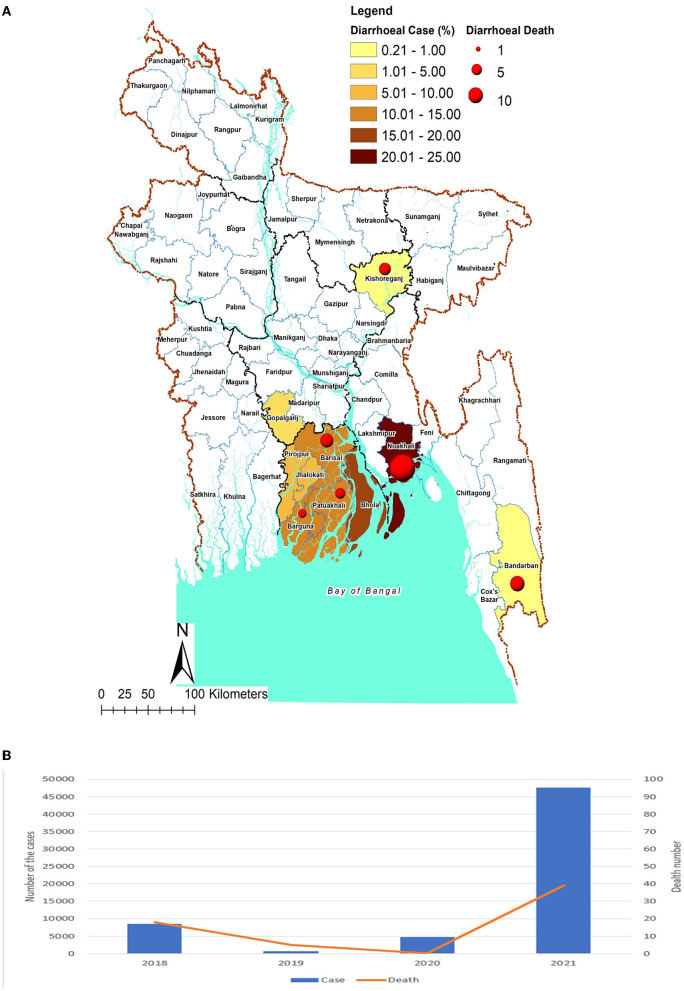
**(A)** The number of cases and death due to diarrhoeal discase outbreaks in different districts/upazila in Bangladesh, 2021. Map source: Banglapedia Bangladesh map digitize and data updated by icddr,b. **(B)** The number of cases and deaths dur to diarrhoeal disease outbreaks in different districts/upazlia in Bangladesh, 2018–2021; Data source from the print and electronic media.

In addition to Barisal, the upsurges were sporadically reported from other eight divisions of Bangladesh including Dhaka and Chattogram. A diarrheal outbreak occurred in early May 2021 in Mithamoin upazila of Kishoreganj District in northern Bangladesh, located in the Mymensingh Division, and more than a hundred children and adults were affected by diarrhea and four individuals died as a result of severe dehydration. In Gopalganj in the Dhaka division, the diarrheal upsurge was recorded from April onward. Increased salinity and pollution of the surrounding river “Modhumoti” in the area were considered major sources of infection (15). Noakhali, a district of Chattogram Division faced a similar outbreak in April–May 2021, where 15 deaths due to diarrheal diseases were notified in 20 days. Around 10,000 people were affected by the disease at that time and a majority of the cases were children and the elderly (16). In early June 2021, diarrhea cases also rose in Alikadam Upazila of Bandarban, and six deaths due to AWD were notified within 4 days from the affected area. The health authority of the Bandarban area and the Military patrol team worked together to control the epidemics by ensuring adequate drinking water, water purification tablets, saline, and other essential medicines (17). The Forcibly Displaced Myanmar Nationals (FDMNs) who fled to Bangladesh in 2017 due to the internal conflict in Myanmar resided in Cox's Bazar. In December 2021, approximately 20,000 FDMNs were shifted to Bhasan Char, an isolated island in Hatiya under the Noakhali district. A diarrheal epidemic was also observed in that area, where 1,500 people were infected along with four deaths. The local health authorities confirm that they were able to control the situation in Bhasan Char (18). We also provide a geographical distribution of the 2021 diarrhea outbreak in Bangladesh (Figure 1A). We have searched different reports to find out the morbidity and mortality due to diarrheal diseases across the country for the period between 2018 and 2021. The death counts and hospitalization rates were higher in 2021 in comparison to other years (19–34) (Figure 1B). Rotavirus, adenovirus, and *Vibrio cholerae* were the most common diarrheagenic microorganisms in Bangladesh, regardless of age or location. It is critical to speed up the introduction of rotavirus and cholera vaccines into the national vaccination program, as these vaccines have the potential to considerably lower the burden (35). The intensity of diarrhea is usually noticed more in places adjacent to coastal environments, particularly in Bangladesh's southern coastal areas and north-eastern regions. The northeastern territory is particularly vulnerable owing to the annual occurrence of severe flooding during the rainy season.

The recent cholera epidemics that occurred in South America (36), Asia (37), and sub-Saharan Africa (38) affected millions of people and had a high mortality rate. The World Health Organization (WHO) documented annual cholera incidences globally (39). Although these are mainly focused on official incidents that the affected countries have documented. These reports are believed to be underestimated due to limitations or lack of adequate surveillance systems. In addition, the actual global number of cholera cases may be estimated to be higher than officially reported (40). Because outbreaks are frequently not reported to avoid the risk of travel and trade embargoes against the affected country. In recent diarrheal outbreaks in Bangladesh, analysis of acute diarrhea cases showed *V. cholerae* to be the most commonly identified causative agent (41).

Primary data were not used in this study, so that was one of the primary limitations. One of the strengths was that the combined data presentation did highlight the 2021 diarrhea outbreak, including mortality, so this will create awareness about future outbreaks of diarrhea in Bangladesh.

## Conclusion

Diarrheal diseases occur every year in Bangladesh, but in 2021, the cases and fatality rates exceeded previous reports in some places. *Vibrio cholerae* is usually a cause of diarrheal epidemics and outbreaks in Bangladesh (11); however, due to the lack of microbiological data, we are unable to determine the cause. The establishment of a national surveillance network with enhanced laboratory capacity for early detection and immediate action is key for combating the disease. However, to achieve the target of cholera ending by 2030, different intervention strategies such as improvement of water sanitation and hygiene facilities, immunization including cholera vaccine in the hotspot, household water treatment, and preventive treatment for household contact can play a major role in preventing diarrheal disease. In conclusion, enhanced awareness and alert systems, sustainable surveillance, and epidemiological studies can track trends in diarrheal disease incidence and mortality along with future projections, which will lead to evaluations of different prevention and control strategies.

## Author contributions

The article's first draft was written by AK, MI, and MA. AK, FQ, MA, MI, and ZK contributed to the literature review and manuscript preparation. All authors contributed to the final version by critically reviewing and editing drafts.
